# Radiological Features of Primary Pulmonary Invasive Mucinous Adenocarcinoma Based on 312 Consecutive Patients

**DOI:** 10.1111/crj.13820

**Published:** 2024-08-08

**Authors:** Linlin Qi, Jia Jia, Guochao Zhang, Jianing Liu, Fenglan Li, Jiaqi Chen, Shulei Cui, Sainan Cheng, Liyan Xue, Qi Xue, Jianwei Wang

**Affiliations:** ^1^ Department of Diagnostic Radiology, National Cancer Center/National Clinical Research Center for Cancer/Cancer Hospital Chinese Academy of Medical Sciences and Peking Union Medical College Beijing China; ^2^ Department of Pathology, National Cancer Center/National Clinical Research Center for Cancer/Cancer Hospital Chinese Academy of Medical Sciences and Peking Union Medical College Beijing China; ^3^ Department of Thoracic Surgery, National Cancer Center/National Clinical Research Center for Cancer/Cancer Hospital Chinese Academy of Medical Sciences and Peking Union Medical College Beijing China

**Keywords:** differential diagnosis, lung, mucinous adenocarcinoma, x‐ray computed tomography

## Abstract

**Background:**

The aim of this study is to investigate the radiological features of primary pulmonary invasive mucinous adenocarcinoma (IMA) in a relatively large population to help improve its further understanding and its accuracy of initial diagnosis.

**Methods:**

This retrospective study included consecutive patients with pathologically confirmed primary pulmonary IMA from January 2019 to December 2021. According to tumor morphology, IMAs were divided into regular nodule/mass, irregular, and large consolidative types. According to tumor density, IMAs were divided into solid, halo, part‐solid, pure ground‐glass, and cystic types. ANOVA, chi‐square, or Fisher exact tests were used to analyze the differences in radiological and clinicopathological characteristics of IMA according to morphological and density subtypes.

**Results:**

We analyzed 312 patients. Pulmonary IMA tended to occur in the elderly, with a slightly higher number of women than men. IMA showed a predominance in the lower lobe and adjacent to pleura. IMA of regular nodule/mass, irregular, and large consolidative types accounted for 80.8% (252/312), 13.8% (43/312), and 5.4% (17/312), respectively. Solid, halo, part‐solid, pure ground‐glass, and cystic IMAs accounted for 55.8% (174/312), 28.2% (88/312), 11.2% (35/312), 1.3% (4/312), and 3.5% (11/312), respectively. The lobulated (76.9%), spiculated (63.5%), and air bronchogram (56.7%) signs were common in IMA. Dead branch sign (88.2%), angiogram sign (88.2%), and satellite nodules/skipping lesions (47.1%) were common in large‐consolidative‐type IMA. Kirsten rat sarcoma viral oncogene mutations were common (56.1%), whereas epidermal growth factor receptor mutations were relatively rare (2.3%).

**Conclusions:**

Pulmonary IMA of regular nodule/mass type and solid type were the most common at the initial diagnosis. Detailed radiological features can aid in the differential diagnosis of IMA.

## Graphical abstract

This study systematically summarized the radiological characteristics of primary pulmonary invasive mucinous adenocarcinoma (IMA) in a relatively large population to improve its early diagnosis rate and reduce misdiagnosis rate. Pulmonary IMA showed a predominance in the lower lobe and adjacent to pleura. Pulmonary IMA of regular nodule/mass, irregular, and large consolidative types accounted for 80.8% (252/312), 13.8% (43/312), and 5.4% (17/312), respectively. Solid, halo, part‐solid, pure ground‐glass, and cystic IMAs accounted for 55.8% (174/312), 28.2% (88/312), 11.2% (35/312), 1.3% (4/312), and 3.5% (11/312), respectively.

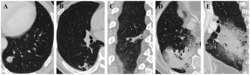

## 1 Introduction

Primary pulmonary invasive mucinous adenocarcinoma (IMA) is a distinct subtype of adenocarcinoma according to the current World Health Organization classification of lung cancer [1, 2, 3]. It is rare, accounting for approximately 3%–10% of all lung adenocarcinomas [3]. Pulmonary IMA with goblet cell or columnar cell morphology with abundant intracytoplasmic mucin, based on the percentage of mucinous component, can be divided into two subtypes: pure mucinous type (invasive mucinous component > 90%) and mixed mucinous/non‐mucinous type (invasive non‐mucinous component ≥ 10%) [3, 4, 5]. Pulmonary IMAs have different characteristics than non‐mucinous adenocarcinomas (NMA) in terms of clinical, histological, genetic, and radiological features. As a subtype of lung adenocarcinoma, early diagnosis and treatment are the most important means of improving the prognosis of patients with IMA. However, in clinical practice, we found that pulmonary IMAs have various morphological subtypes (such as regular‐nodule/mass, irregular, and large‐consolidative/pneumonic‐like patterns), various density subtypes (such as solid, halo, part‐solid, pure ground‐glass, and cystic types), and various internal features. Due to the diverse radiological manifestations of IMA, as well as its overlap with pneumonia and NMA imaging, IMA imaging diagnosis is very challenging at the initial presentation. Owing to the low incidence rate of pulmonary IMA, few studies with a relatively large population have systematically summarized its radiological characteristics.

Therefore, the purpose of this study was to investigate the radiological features of primary pulmonary IMA in a relatively large population to help improve its further understanding and its accuracy of initial diagnosis.

## 2 Methods

### 2.1 Study Population

**FIGURE 1:**
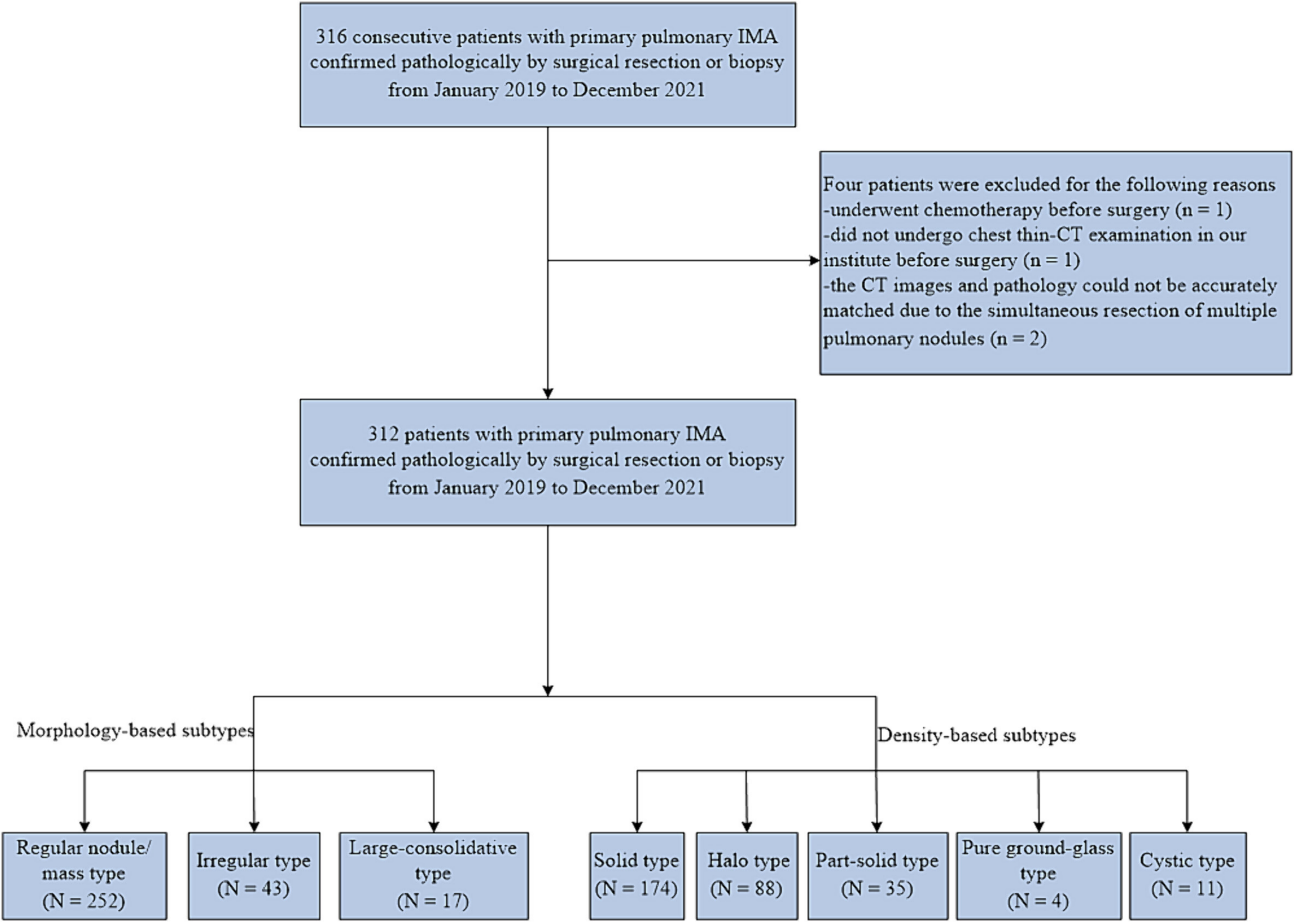
Flowchart showing the inclusion and exclusion criteria and the grouping of patients. IMA = invasive mucinous adenocarcinoma.

We retrospectively reviewed the medical records of 316 consecutive patients with primary pulmonary IMA confirmed pathologically by surgical resection or biopsy at our institution from January 2019 to December 2021. Patients with pulmonary IMA who had not previously received any treatment before operation and who were confirmed to have undergone chest computed tomography (CT) examination within 1 month of diagnosis were eligible. Patients were excluded if they lacked thin‐section chest CT (≤ 1.25 mm) images before diagnosis; they underwent preoperative radiation therapy, chemotherapy, or immunotherapy treatment; their imaging and pathology could not be accurately matched because multiple pulmonary nodules were removed simultaneously; or they lacked complete clinicopathologic data (Figure 1). This retrospective study was approved by the ethics committee of our institution, and the need for informed consent was waived.

### 2.2 CT Image Acquisition

All CT images were acquired using 64‐row spiral CT scanners (LightSpeed VCT, Discovery CT750 HD, Optima CT66 scanner, GE Healthcare, Chicago, IL; IQon Spectral CT, Philips, Amsterdam, The Netherlands). The CT parameters were as follows: tube voltage, 120 kVp and auto mA settings (tube current, approximately 200–350 mA; noise index, 13; pitch, 0.992 or 0.984; rotation time, 0.5 s; thickness, 5 mm). The reconstruction thicknesses were 1.25, 1.0, or 0.8 mm, and the intervals were 0.8 mm using a standard reconstruction algorithm. The enhanced CT scan commenced after a 35 s delay after intravenous injection of approximately 80–90 mL of contrast medium (iodine concentration of 300 mg/mL) using a power injector at a rate of 2.5 mL/s.

### 2.3 Radiological Analysis

Two thoracic radiologists (JWW and LLQ, with 25 and 7 years of experience in chest CT, respectively) independently reviewed the CT images and assessed the following radiological characteristics. Disagreements were resolved by consultation.

**FIGURE 2:**
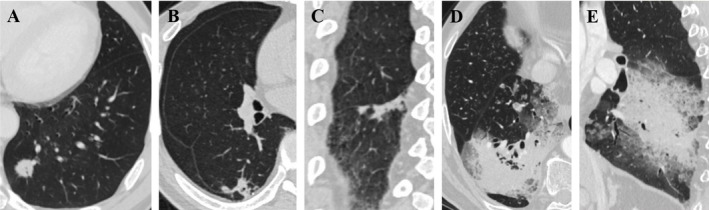
Pulmonary invasive mucinous adenocarcinoma (IMA) with three morphological subtypes. (A) Regular nodule/mass type. (B,C) Irregular type (from a same patient). (D,E) Large consolidative type (from a same patient).

Pulmonary IMAs were divided into three subtypes according to tumor CT morphology: regular nodule/mass, irregular, and large consolidative types. The regular nodule/mass type was defined when the tumor presented as a round or round‐like nodule or mass on CT multiplanar reconstruction (MPR) images, regardless of contour, border, or density characteristics. The large consolidative type was defined as diffuse consolidation (1) involving one or more lobes; (2) involving the inner, middle, and outer zones of a lobe; or (3) involving more than a quarter of the area of a lobe. The irregular type refers to tumors that could not be classified as the regular nodule/mass type or large consolidative type, and its shape was extremely irregular on MPR images (Figure 2).

**FIGURE 3:**
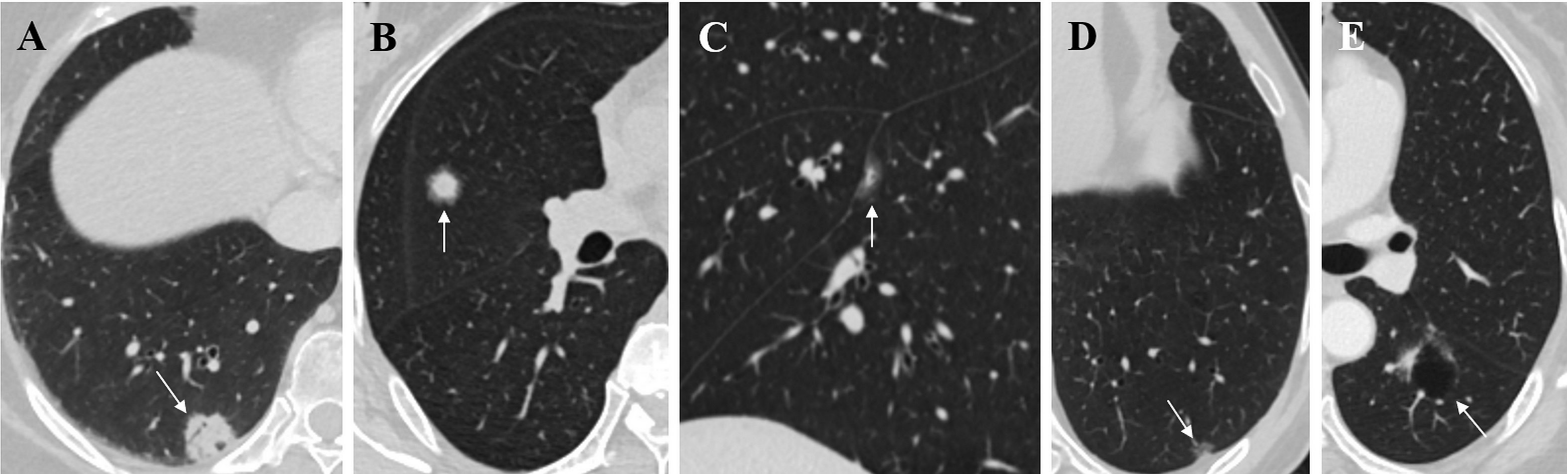
Pulmonary invasive mucinous adenocarcinoma (IMA) with five density subtypes. (A) Solid type. (B) Halo type. (C) Part‐solid type. (D) Pure ground‐glass type. (E) Cystic type.

IMAs were divided into five subtypes according to tumor CT density: solid, halo, part‐solid, pure ground‐glass, and cystic types. The solid type was defined as a lesion containing only solid components. The halo type was defined as a lesion with surrounding ground‐glass opacity, and the ratio of the maximum diameter of the ground‐glass component to the whole lesion was < 20%. The part‐solid type was defined as a lesion with a combination of both ground‐glass and solid components, and the ratio of the maximum diameter of the ground‐glass component to the whole lesion was ≥ 20%. The pure ground‐glass type was defined as lesions containing only ground‐glass components without solid components. The cystic type was defined as a lesion mainly composed of a cystic cavity, surrounded by ground‐glass or solid components, and the ratio of the maximum diameter of the cystic cavity to that of the entire lesion was ≥ 50%. If the lesion contained both cystic cavity and ground glass, the dominant component (≥ 50% of the entire lesion) was used to define the type of lesion (Figure 3).

**FIGURE 4:**
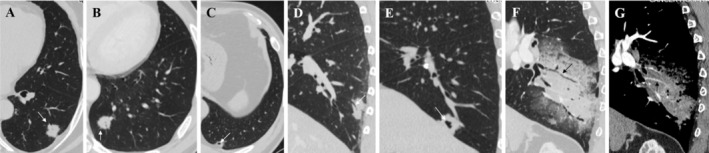
Morphological/internal features of pulmonary invasive mucinous adenocarcinoma on CT images. (A,E) Lobulated sign. (A,B) Spiculated sign. (C) Vacuole sign, with the diameter of air‐containing space ≤ 5 mm. (D) Air bronchogram sign, referring to the smooth bronchus passing through the consolidation area. (E) Cystic cavity, with the diameter of air‐containing space >5 mm. (A,C,D,E) Pleural attachment. (F) Dead branch sign, referring to the dilatation, stiffness, or narrowing of bronchi passing through the consolidation area. (G) Angiogram sign, named after the obviously enhanced branching pulmonary vessels passing through the low‐density consolidation area on the enhanced CT.

Radiological features included lesion location, mean diameter, mean density, morphological subtype, density subtype, and morphologic/internal features (including lobulated sign, spiculated sign, vacuole sign, air bronchogram sign, cystic cavity, pleural attachment, dead branch sign, and angiogram sign) (Figure 4). The mean diameter was defined as the average of the maximal length and maximal orthogonal diameter on the maximum CT sections. The mean density was defined as the average CT value of the tumor on the maximum sections of the CT lung window, avoiding the blood vessels, bronchi, and cysts. Air bronchogram sign was defined as air‐filled bronchi within lesions. Dead branch sign was defined as an irregular air bronchogram that appeared as dilatation, stiffness, or narrowing of bronchi within lesions, shaped like dead branches. Angiogram sign was defined as the obviously enhanced branching pulmonary vessels passing through the low‐density consolidation area on the enhanced CT. Notably, the above radiological features were analyzed using MPR images. Images were viewed both at the lung window (window width, 1600 HU; level, −600 HU) and at the mediastinal window (window width, 350 HU; level, 40 HU).

In addition to the major lesion, the presence of satellite nodules or skipping lesions was also assessed. The above radiological features of the major lesion were reviewed and recorded in detail, whereas satellite nodules or skipping lesions were only recorded as “yes or no.”

### 2.4 Clinical and Pathological Analyses

Clinical data included age, sex, clinical symptoms, smoking history, malignancy history, diagnosis method, and operation time.

The pathology slides were reviewed by two pathologists (LYX and JJ, with 21 and 7 years of experience in the pathological diagnosis of thoracic tumors, respectively), following the 5th edition of the World Health Organization classification of thoracic tumors [3] and the 8th edition of the American Joint Committee on Cancer Staging System for non–small cell lung cancer [6]. The degree of pleural invasion was evaluated using elastic staining. Moreover, molecular analyses of the mutation status of the epidermal growth factor receptor and Kirsten rat sarcoma viral oncogene were examined with polymerase chain reaction–based amplification refractory mutation system using the Human EGFR Gene Mutations Detection Kit (Beijing ACCB Biotech Ltd., Beijing, China).

### 2.5 Statistical Analysis

Continuous data with the normal distribution are expressed as mean ± standard deviation; otherwise, the medians (range) are presented. Categorical data are presented as numbers (percentages). The differences in radiological and clinicopathological characteristics of IMA with different morphological and density subtypes were assessed with univariable analysis. Continuous variables were compared by the ANOVA test. Categorical variables were compared by the chi‐squared test or Fisher's exact test. *p* < 0.05 was considered to indicate statistical significance. All statistical tests were performed using SPSS Version 25.0 (IBM Corp., Armonk, NY).

## 3 Results

### 3.1 Patient Characteristics

Among the 316 patients, four were excluded because they underwent chemotherapy before surgery (*n* = 1), they did not undergo thin‐section chest CT examination at our institute before surgery (*n* = 1), or the CT images and pathology could not be accurately matched owing to the simultaneous resection of multiple pulmonary nodules (*n* = 2) (Figure 1). Finally, 312 patients with primary pulmonary IMA confirmed by surgical resection or biopsy were enrolled.

The radiological and clinicopathological characteristics of patients with pulmonary IMA are described in Table 1. Clinically, pulmonary IMA tended to occur in the elderly (mean age ± standard deviation, 59 ± 10 years; age range, 20–82 years), with slightly more women than men (1.38:1). Only 24.4% of the patients with IMA had a history of smoking. 81.7% (255/312) of pulmonary IMAs were discovered incidentally during physical examination, and only 18.3% (57/312) had clinical symptoms including cough (49/57), expectoration (29/57), bloody sputum (6/57), chest discomfort (6/57), fever (4/57), and hemoptysis (1/57).

**TABLE 1:** Characteristics of the 312 patients with pulmonary invasive mucinous adenocarcinoma.

Clinical characteristics	
Age (years)	59 ± 10
Gender	
Female	181 (58.0)
Male	131 (42.0)
Smoking history	
Current/ever	76 (24.4)
Never	236 (75.6)
Malignant history	
Yes	22 (7.1)
No	290 (92.9)
Clinical symptoms	
No	255 (81.7)
Yes	57 (18.3)
Cough	49 (86.0)
Expectoration	29 (50.9)
Bloody sputum	6 (10.5)
Hemoptysis	1 (1.8)
Fever	4 (7.0)
Chest discomfort	6 (10.5)

Radiological characteristics	
Tumor location	
Right upper lobe	42 (13.5)
Right middle lobe	25 (8.0)
Right lower lobe	97 (31.1)
Left upper lobe	44 (14.1)
Left lower lobe	104 (33.3)
Mean diameter of tumor (mm)	18.7 (7.6–131.9)
Mean density of tumor (HU)	−79 (−631–103)
Classification by tumor morphology	
Regular nodule/mass type	252 (80.8)
Irregular type	43 (13.8)
Large consolidative type	17 (5.4)
Classification by tumor density	
Solid type	174 (55.8)
Halo type	88 (28.2)
Part‐solid type	35 (11.2)
Pure ground‐glass type	4 (1.3)
Cystic type	11 (3.5)
Morphologic/internal features	
Lobulated sign	240 (76.9)
Spiculated sign	198 (63.5)
Vacuole sign	49 (15.7)
Air bronchogram sign	177 (56.7)
Cystic cavity	32 (10.3)
Pleural attachment	241 (77.2)
Dead branch sign	15 (4.8)
Angiogram sign	15 (4.8)
Satellite nodules/skipping lesions	
Yes	18 (5.8)
No	294 (94.2)

Pathological characteristics	
Maximum diameter of tumor (mm)	17 (3–12)
Pathological staging	
T staging	
T1	228 (73.1)
T2	48 (15.4)
T3	15 (4.8)
T4	13 (4.2)
Tx	8 (2.5)
N staging	
N0	286 (91.7)
N1	10 (3.2)
N2	7 (2.2)
Nx	9 (2.9)
Spread‐through‐air‐spaces (STAS)	
Yes	52 (16.7)
No	260 (83.3)
Pleural invasion	
Yes	21 (6.7)
No	277 (88.8)
Unassessed	14 (4.5)
Genetic testing	221
KRAS (+)	124 (56.1)
EGFR (+)	5 (2.3)

*Note:* Values are expressed as number (percentage), mean ± standard deviation, or median (range). Tx refers to patients who did not obtain pathological T staging because they did not undergo surgery or elastic staining. Nx refers to patients who did not obtain pathological N staging because they did not undergo systematic mediastinal lymphadenectomy.

Abbreviations: EGFR = epidermal growth factor receptor, KRAS = Kirsten rat sarcoma viral oncogene.

Radiologically, among the 312 pulmonary IMAs, 64.4% were located in the lower lobe, and 77.2% were pleura attachments. The mean diameter of the tumor was 25.3 ± 20.0 mm, with a median of 18.7 mm (range, 7.6–131.9 mm). Morphologically, the 312 pulmonary IMAs consisted of 252 regular nodule/mass type (80.8%), 43 irregular type (13.8%), and 17 large consolidative type (5.4%) (Figures 1 and 2). Based on the tumor density on CT, the 312 pulmonary IMAs consisted of 174 solid (55.8%), 88 halo (28.2%), 35 part‐solid (11.2%), 4 pure ground‐glass (1.3%), and 11 cystic (3.5%) types (Figures 1 and 3). Moreover, the lobulated sign (76.9%), spiculated sign (63.5%), air bronchogram sign (56.7%), and pleural attachment (77.2%) were common CT features of pulmonary IMA. Eighteen (5.8%) pulmonary IMAs had satellite nodules or skipping lesions on CT, including 8/17 large consolidative types, 8/252 regular nodule/mass types, and 2/43 irregular types.

Pathologically, 73.1% of the patients with IMA were in stage T1, 24.4% were in stage T2–4, and 2.5% did not obtain pathological T staging because they did not undergo surgery or elastic staining (their clinical T staging included three cases of stage T4, one case of stage T2b, two cases of stage T1c, and two cases of stage T1b). Only 6.7% of IMA lesions invaded the pleura. 91.7% of patients had no lymph node metastasis. Two patients had pleural metastasis at the time of the first diagnosis. Spread‐through‐air‐spaces was detected in 16.7% of IMAs. In addition, 221 patients underwent genetic testing, of which Kirsten rat sarcoma viral oncogene (KRAS) mutations accounted for 56.1%, whereas epidermal growth factor receptor (EGFR) mutations accounted for only 2.3%.

### 3.2 Radiological and Clinicopathological Characteristics of Pulmonary IMA With Different Morphological Subtypes

**FIGURE 5:**
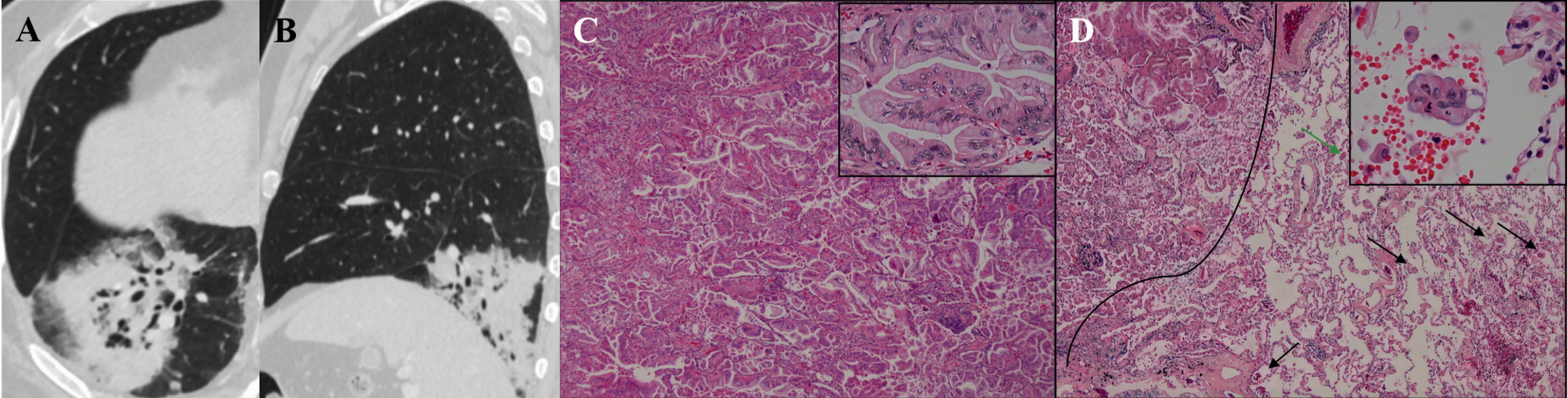
Large‐consolidative‐type IMA in a 73‐year‐old male. Axial CT image (A) and sagittal CT image (B) show a large‐consolidative‐type lesion with the diameter of 105.2 × 60.5 mm in right lower lobe. (C) Low magnification (hematoxylin eosin, 40×) of the histologic specimen shows that the lesion is IMA with papillary and micropapillary patterns. High magnification (hematoxylin eosin, 400×) shows that the tumor cells have abundant mucus and the nucleus is located at the base, with moderate atypia (black box in the upper right corner). (D) Low magnification (hematoxylin eosin, 40×) shows that STAS (black arrow) are seen in the surrounding alveolar cavity of the tumor; the STAS at the green arrow is observed at the high magnification (hematoxylin eosin, 400×), appearing as clusters of tumor cells arranged in a micropapillary structure (black box in the upper right corner). IMA = invasive mucinous adenocarcinoma; CT = computed tomography; STAS = spread‐through‐air‐spaces.

The distribution of mean diameter (*p* < 0.001); mean density (*p* = 0.002); density subtypes (*p* = 0.01); lobulated (*p* < 0.001), spiculated (*p* = 0.01), vacuole (*p* = 0.001), air bronchogram (*p* < 0.001), dead branch (*p* < 0.001), and angiogram (*p* < 0.001) signs; cystic cavity (*p* = 0.03); pleural attachment (*p* = 0.008); satellite nodules/skipping lesions (*p* < 0.001); pathological T (*p* < 0.001) and N (*p* = 0.02) staging; spread‐through‐air‐spaces (*p* < 0.001); and pleural invasion (*p* = 0.04) differed among the regular nodule/mass, irregular, and large consolidative groups (Table 2). Lobulated, spiculated, and air bronchogram signs and pleural attachment were more common in regular nodule/mass‐type IMA, accounting for 79.8% (201/252), 65.9% (166/252), 49.2% (124/252), and 73.8% (186/252), respectively. Dead branch sign, angiogram sign, and satellite nodules/skipping lesions were more common in the large‐consolidative‐type IMA, accounting for 88.2% (15/17), 88.2% (15/17), and 47.1% (8/17), respectively. Pathologically, at initial diagnosis, most patients with large‐consolidative‐type IMA were in stage T3–4 (88.2%), whereas most patients with regular/irregular‐type IMA were in stage T1–2 (93.2%). The incidence of spread‐through‐air‐spaces was relatively higher in large‐consolidative‐type IMA, accounting for 35.3% (6/17) (Figure 5).

**TABLE 2:** Radiological and clinicopathological characteristics of pulmonary invasive mucinous adenocarcinoma with different morphological subtypes.

Morphological types	Regular nodule/mass type	Irregular type	Large consolidative type	*p*
Number of patients	252	43	17	NA
Clinical characteristics				
Age (years)	59 ± 10	60 ± 9	60 ± 11	0.66^a^
Gender				0.11^b^
Female	139	30	12	
Male	113	13	5	
Radiological characteristics				
Tumor location				0.15^b^
Upper lobe	163	24	14	
Middle or lower lobe	89	19	3	
Mean diameter of tumor (mm)	20.3 ± 11.8	30.5 ± 11.9	86.1 ± 28.9	< 0.001^a^
Mean density of tumor (HU)	−151 ± 180	−128 ± 153	4.2 ± 59.3	0.002^a^
Classification by tumor density				0.01^c^
Solid type	147	23	4	
Halo type	63	12	13	
Part‐solid type	28	7	0	
Pure ground‐glass type	4	0	0	
Cystic type	10	1	0	
Morphologic/internal features				
Lobulated sign	201	33	6	< 0.001^b^
Spiculated sign	166	27	5	0.01^b^
Vacuole sign	34	9	8	0.001^b^
Air bronchogram sign	124	39	15	< 0.001^b^
Cystic cavity	22	5	5	0.03^c^
Pleural attachment	186	38	17	0.008^b^
Dead branch sign	0	0	15	< 0.001^c^
Angiogram sign	0	0	15	< 0.001^c^
Satellite nodules/skipping lesions				< 0.001^c^
Yes	8	2	8	
No	244	41	9	
Pathological characteristics				
Pathological staging				
T staging				< 0.001^c^
T1–2	236	39	1	
T3–4	11	2	15	
Tx	5	2	1	
N staging				< 0.02^c^
N0	232	42	12	
N1–2	11	1	4	
Nx	9	0	1	
Spread‐through‐air‐spaces (STAS)				< 0.001^b^
Yes	41	5	6	
No	211	38	11	
Pleural invasion				0.04^c^
Yes	17	2	2	
No	227	38	12	
Unassessed	8	3	3	
KRAS				0.77^b^
(+)	98	17	9	
(−)	80	12	5	

*Note:* Values are expressed as number, mean ± standard deviation, or median (range). Tx refers to patients who did not obtain pathological T staging because they did not undergo surgery or elastic staining. Nx refers to patients who did not obtain pathological N staging because they did not undergo systematic mediastinal lymphadenectomy.

Abbreviation: KRAS = Kirsten rat sarcoma viral oncogene.

^a^ ANOVA test.

^b^ Chi‐square test.

^c^ Fisher's exact test.

### 3.3 Radiological and Clinicopathological Characteristics of Pulmonary IMA With Different Density Subtypes

The distribution of the mean diameter (*p* = 0.01); mean density (*p* < 0.001); morphological classification (*p* = 0.01); lobulated (*p* < 0.001), spiculated (*p* < 0.001), dead branch (*p* < 0.001), and angiogram (*p* < 0.001) signs; cystic cavity (*p* < 0.001); pleural attachment (*p* = 0.01); pathological T staging (*p* = 0.04); and spread‐through‐air‐spaces (*p* = 0.002) differed among the solid‐type, halo‐type, part‐solid‐type, pure‐ground‐glass‐type, and cystic‐type IMA (Table 3). Halo sign was most common in large‐consolidative‐type IMA (13/17, 76.5%).

**TABLE 3:** Radiological and clinicopathological characteristics of pulmonary invasive mucinous adenocarcinoma with different density subtypes.

Density types	Solid type	Halo type	Part‐solid type	Pure ground‐glass type	Cystic type	*p*
Number of patients	174	88	35	4	11	NA
Clinical characteristics						
Age (years)	59 ± 10	60 ± 10	58 ± 10	62 ± 9	59 ± 13	0.72^a^
Gender						0.07^b^
Female	91	61	21	3	5	
Male	83	27	14	1	6	
Radiological characteristics						
Tumor location						0.10^b^
Upper lobe	112	59	17	3	10	
Middle or lower lobe	62	29	18	1	1	
Mean diameter of tumor (mm)	24.0 ± 13.7	30.1 ± 29.8	18.9 ± 8.2	10.8 ± 3.1	31.9 ± 28.0	0.01^a^
Classification by tumor morphology						0.01^b^
Regular nodule/mass type	147	63	28	4	10	
Irregular type	23	12	7	0	1	
Large consolidative type	4	13	0	0	0	
Morphologic/internal features						
Lobulated sign	154	58	19	1	8	< 0.001^b^
Spiculated sign	138	48	7	0	5	< 0.001^b^
Vacuole sign	30	17	3	0	1	0.63^b^
Air bronchogram sign	96	59	16	2	5	0.15^b^
Cystic cavity	14	6	1	0	11	< 0.001^b^
Pleural attachment	145	64	21	2	9	0.01^b^
Dead branch sign	2	13	0	0	0	< 0.001^b^
Angiogram sign	2	13	0	0	0	< 0.001^b^
Satellite nodules/skipping lesions						0.34^b^
Yes	10	8	0	0	0	
No	164	80	35	4	11	
Pathological characteristics						
Pathological staging						
T staging						0.04^b^
T1–2	155	74	34	4	9	
T3–4	13	14	0	0	1	
Tx	6	0	1	0	1	
N staging						0.56^b^
N0	158	79	35	4	10	
N1–2	11	5	0	0	0	
Nx	5	4	0	0	1	
Spread‐through‐air‐spaces (STAS)						0.002^b^
Yes	37	11	0	0	4	
No	137	77	35	4	7	
Pleural invasion						0.94^b^
Yes	15	5	1	0	0	
No	150	79	33	4	11	
Unassessed	9	4	1	0	0	
KRAS						0.81^b^
(+)	68	37	12	1	6	
(−)	57	26	11	1	2	

*Note:* Values are expressed as number or mean ± standard deviation. Tx refers to patients who did not obtain pathological T staging because they did not undergo surgery or elastic staining. Nx refers to patients who did not obtain pathological N staging because they did not undergo systematic mediastinal lymphadenectomy.

Abbreviation: KRAS = Kirsten rat sarcoma viral oncogene.

^a^ ANOVA test.

^b^ Fisher's exact test.

## 4 Discussion

Primary pulmonary IMA is often misdiagnosed at initial presentation, potentially leading to missing the best opportunity for surgical resection. We investigated the radiological characteristics of primary pulmonary IMA in a relatively large population to help improve its further understanding and its accuracy of initial diagnosis. The dominant locations of the lesions were the lower lobes and adjacent to the pleura. At the first diagnosis, 80.8% of the lesions were of regular nodule/mass type. In terms of tumor density on CT, more than half (55.8%) of the lesions were solid, and pure ground‐glass and cystic types were rare. The lobulated (76.9%), spiculated (63.5%), and air bronchogram (56.7%) signs were common in IMA. Dead branch sign (88.2%), angiogram sign (88.2%), and satellite nodules/skipping lesions (47.1%) were common in large‐consolidative‐type IMA. Pathologically, patients tended to have early‐stage IMA at the initial diagnosis. Compared with the regular nodule/mass and irregular types, higher T and N stages were found in large‐consolidative‐type lesions. KRAS mutations were common oncogenic driver mutations (56.1%), whereas EGFR mutations were relatively rare (2.3%).

Clinically, pulmonary IMA tended to occur in the elderly, with slightly more women than men, which is consistent with previous studies [5, 7, 8, 9, 10, 11, 12]. There was no significant association between smoking and IMA, which is also similar to previous studies [5, 7, 8, 9]. However, Azari et al. [11] showed that 14 of 19 patients with IMA had a smoking history. Most patients with IMA had no obvious symptoms. Cough and expectoration were the most common respiratory symptoms, whereas fever was relatively rare, which was helpful in distinguishing IMA from infectious pneumonia. Additionally, among the 17 cases of large‐consolidative‐type IMA, only two patients (2/17, 11.8%) had fever symptom in this study, which was helpful in distinguishing pneumonia‐type IMA and pneumonia.

Previous studies investigated the imaging features of IMA [7, 9, 12, 13, 14, 15, 16, 17]; however, they all had limitations, including a relatively small sample size [7, 9, 15, 16], only including pneumonic‐type IMA [13, 14, 15], or focusing on IMA prognosis rather than CT features [12, 17], and detailed reports of the radiological features of IMA are relatively rare. Our findings will, therefore, help improve the overall impression of IMA and the accuracy of its initial diagnosis. We found that the dominant locations of the IMA were the lower lobe and adjacent to the pleura, which is similar to previous studies [7, 10, 11, 12]. IMA showed a lower lobe and peripheral predominance, which may be due to gravity.

In terms of tumor morphology on CT, regular nodule/mass type was the most common subtype, accounting for 80.8%, whereas the irregular type and large‐consolidative type accounted for only 13.8% and 5.4%, respectively. Nie et al. [9], Wang et al. [12], and Watanabe et al. [16] reported that the solitary type and pneumonic type accounted for 79.4% (54/68) and 20.6% (14/68), 91.8% (291/317) and 8.2% (26/317), and 75% (30/40) and 25% (10/40) of surgically resected IMA, respectively. Yoon et al. [17] classified the IMA undergoing surgical resection into two subtypes, namely, nodular and consolidative types, accounting for 75.2% (91/121) and 24.8% (30/121), respectively. The regular nodule/mass type in this study was equivalent to the solitary type in the aforementioned studies, and the large consolidative type was equivalent to the pneumonic type. Although the definition of the irregular type was controversial in different studies, the irregular type can also be classified as the localized‐pneumonic type. For the regular nodule/mass‐type IMA, it was difficult to radiologically differentiate it from NMA. We found that lobulated sign (201/252, 79.8%), spiculated sign (166/252, 65.9%), air bronchogram sign (124/252, 49.2%), and pleural attachment (186/252, 73.8%) were common in the regular nodule/mass‐type IMA. Irregular‐type IMA was easily misdiagnosed as focal pneumonia. We found that air bronchogram sign (39/43, 90.7%), lobulated sign (33/43, 76.7%), and spiculated sign (27/43, 62.8%) were common in irregular‐type IMA. Previous studies also showed that irregular air bronchogram, lobulated sign, and spiculated sign were frequently present in malignant nodules than focal pneumonia [14, 15]. Large‐consolidative‐type IMA was easily misdiagnosed as pneumonia, interstitial lung disease, diffuse alveolar hemorrhage, and other benign lung lesions. The possibility of the large‐consolidative‐type (also named pneumonia‐type) IMA should be highly suspected when the following signs appear on chest CT: dead branch sign, angiogram sign, halo sign, satellite nodules/skipping lesions, and relatively lower enhancement [13, 14]. Additionally, the respiratory symptoms and hemogram can help the differential diagnosis between the IMA and inflammatory or infectious process. We found that the majority of IMA patients (81.7%) had no clinical symptoms. Zhang et al. [14] reported that the patients with pneumonic‐type IMA showed a higher prevalence of no symptoms than those with infectious pneumonia; fever, elevation of white blood cell count, and elevation of C‐reactive protein level were more commonly observed in patients with infectious pneumonia than those with pneumonic‐type IMA. Moreover, previous studies reported that compared with the solitary‐type IMA, the large‐consolidative‐type (or pneumonia‐type) IMA showed a significantly poorer prognosis [9, 12, 17, 18, 19]. The resectable IMA patients had a better prognosis than patients with NMA [20, 21].

Based on the tumor density on CT, 55.8% of IMAs were solid type, 28.2% were halo type, 11.2% were part‐solid type, 1.3% were pure ground‐glass type, and 3.5% were cystic type. Kim et al. [7] reported that early‐stage IMAs presented as a solid (54.9%), part‐solid (38.5%), and pure ground‐glass (6.6%) nodule. We speculate that Kim et al. classified halo‐type nodules as part‐solid nodules. We found that the halo sign was a characteristic manifestation of IMA. The formation of a halo sign may be associated with the production of mucus by tumor cells, lepidic‐predominant adenocarcinoma at the edge of the tumor, or its spread through the airway [16].

Pathologically, although primary pulmonary IMA was thought to be diagnosed at an advanced inoperable stage, we observed that patients with IMA tended to have an early T stage at initial diagnosis, and lymph node metastasis was relatively rare. Compared with the regular nodule/mass and irregular types, a higher T stage was found in the large‐consolidative‐type IMA, similar to previous studies [9, 18]. Additionally, 47.1% of the large‐consolidative‐type IMA showed satellite nodules or skipping lesions, which may be related to tumor cells spreading to other lung tissues along small airways or aberrant mucin expression [16, 22]. Regarding the molecular features of IMA, KRAS mutations were common oncogenic driver mutations (56.1%), whereas EGFR mutations were relatively rare (2.3%), similar to previous studies [8, 17, 22, 23].

This study has several limitations. First, it was a retrospective, single‐center study with inevitable inclusion bias and heterogeneous CT scan protocols. Second, most of the enrolled IMAs were confirmed by surgical resection, whereas the number of IMA confirmed by biopsy was very small because most biopsy cases were only confirmed as lung adenocarcinoma, not IMA; thus, the number of large‐consolidative‐type IMAs was relatively small in this study. Finally, the prognosis of IMA was not investigated and will be considered in a future study.

## 5 Conclusions

In conclusion, using a large population of patients with IMA, we described the radiological and clinicopathological characteristics of this rare subtype of adenocarcinoma. Our findings can be used to improve the early diagnosis and reduce the misdiagnosis of IMA.

## Author Contributions

All authors had full access to the data in the study and take responsibility for the integrity of the data and the accuracy of the data analysis. Conceptualization: Jianwei Wang and Linlin Qi. Methodology: Jianwei Wang and Linlin Qi. Investigation: Linlin Qi, Jia Jia, Guochao Zhang, Jianing Liu, Fenglan Li, Jiaqi Chen, Shulei Cui, and Sainan Cheng. Formal analysis: Linlin Qi, Jia Jia, and Jianwei Wang. Resources: Linlin Qi, Jia Jia, Guochao Zhang, Liyan Xue, Qi Xue, and Jianwei Wang. Writing – original draft: All authors. Writing – review and editing: Linlin Qi and Jianwei Wang. Visualization: Linlin Qi and Jianwei Wang. Supervision: Jianwei Wang. Funding acquisition: Jianwei Wang, Linlin Qi and Qi Xue.

## Conflicts of Interest

The authors declare no conflicts of interest.

## Data Availability

Research data are not shared.
